# A research proposal testing a new model of ambulation activity among long-term care residents with dementia/cognitive impairment: the study protocol of a prospective longitudinal natural history study

**DOI:** 10.1186/s13104-019-4585-5

**Published:** 2019-09-03

**Authors:** Mary Elizabeth Bowen, Meredeth A. Rowe, Ming Ji, Pamela Cacchione

**Affiliations:** 1 School of Nursing, University of Delaware, STAR Tower, 100 Discovery Blvd., Newark, DE 19713 USA; 2 Corporal Michael J. Crescenz VA Medical Center, 3900 Woodland Ave., Philadelphia, PA 19104 USA; 3 College of Nursing, University of South Florida, 12901 Bruce B. Downs Blvd, MDC Box 22, Tampa, FL 33612 USA; 4 School of Nursing, University of Pennsylvania, 418 Curie Boulevard, Philadelphia, PA 19104 USA

## Abstract

**Background:**

Excessive and patterned ambulation is associated with falls, urinary tract infections, co-occurring delirium and other acute events among long-term care residents with cognitive impairment/dementia. This study will test a predictive longitudinal data model that may lead to the preservation of function of this vulnerable population.

**Methods/design:**

This is a single group, longitudinal study with natural observations. Data from a real-time locating system (RTLS) will be used to objectively and continuously measure ambulation activity for up to 2 years. These data will be combined with longitudinal acute event and functional status data to capture patterns of change in health status over time. Theory-driven multilevel models will be used to test the trajectories of falls and other acute conditions as a function of the ambulation activity and demographic, functional status, gait quality and balance ability including potential mediation and/or moderation effects. Data-driven machine learning algorithms will be applied to run screening of the high dimensional RTLS data together with other variables to discover new and robust predictors of acute events.

**Discussion:**

The findings from this study will lead to the early identification of older adults at risk for falls and the onset of acute medical conditions and interventions for individualized care.

## Background and significance

A primary goal in long-term care is to stabilize and support physical function to prevent functional decline. Two de-stabilizing events that threaten this goal are falls and acute health changes [e.g., urinary tract infections (UTIs), pneumonia, delirium, upper respiratory infections] that require hospitalization. These are associated with declines in functional status, reduced quality of life and even death [1]. Despite years of research and implemented fall prevention programs, 50% of the 1.6 million nursing home (NH) residents in the U.S. fall each year; 10% of these sustain significant injury [2] and 1800 die as a result of a fall [1]. One factor that contributes to this problem is the inability to identify NH residents that are at the highest risk for falls. The majority of fall assessment tools rate almost all NH residents in the high risk category—making it difficult to apply costly fall-prevention interventions such as extra staffing/supervision. Furthermore, the administration of fall instruments are episodic at best and this strategy can easily miss changes in fall risk between measurements. Fall risk typically includes a falls history (Morse scale) [3] and periodic gait and balance risk assessment by various paper and pencil assessment tools [4, 5]. However, most residents in long-term care have a history of falls (mean 1.7 falls per bed/annually) [6] and gait and balance impairments. The timing of these fall assessments vary by NH, but are typically administered on admittance, quarterly, and/or every 6 months [7–9]. In addition, these assessments are usually administered outside of the natural environment—e.g., in controlled conditions in front of a clinical observer which may vary results [10, 11]. While day-to-day changes in gait can indicate an increased risk for falls, health care staff are unable (and not trained) to continuously observe and objectively measure the quality and quantity of ambulation among long-term care residents to determine if/when ambulation patterns change for each resident. It would also be difficult to implement continuous observations by staff as these are time-consuming. Thus, there is a critical need for more objective, continuous measures of ambulation that automatically ‘observe’ the resident’s day-to-day activities in their natural environment [7, 12, 13]. Furthermore these continuous measures must be combined with ongoing data analysis to detect a change and report that change to health care staff.

Continuous measures of ambulation may also aid in earlier detection of acute health changes such as pneumonia, UTIs and upper respiratory infections, which are relatively common in later life. An estimated 100–300 cases per 1000 NH residents require hospitalization for pneumonia each year; these residents have a 1-year cumulative mortality rate of 50% [14]. In NHs, upper respiratory infections are the most common infection followed by UTIs [15, 16]. UTIs are the most common cause of hospitalization for a bacterial infection [17]. The prevalence of UTIs range from 0.6 to 21.8% and its incidence between 0.3 and 0.8 cases per 1000 resident care days [18]. About 1/3 of UTI’s in NHs are missed, misdiagnosed or improperly treated [19]. This may be because signs and symptoms of infection are missed in this population and some residents with cognitive impairment (CI)/dementia may not show typical signs or symptoms of infection, which largely manifest as changes in functional status [20]. Furthermore, residents with CI/dementia can have a limited ability to verbally express typical UTI symptoms including such as pain and bladder urgency, frequency, and are unable to recognize symptoms such as increased confusion and fever [21]. Older adults, and the minimally trained staff that provide the majority of daily care, may not be able to differentiate mild from more worrisome symptoms in other common illnesses, such as upper respiratory infections and pneumonia, since these presentations change with aging. Additional staff training of certified nursing assistants (CNAs), who provide the majority of direct care to residents in NHs, may address some of these issues [22]. Though required training varies by state—CNAs receive at least 75 initial training hours with a minimum of 16 clinical hours plus 12 annual in-service training hours [23].

The delay in diagnosis and proper care is associated with higher rates of hospitalization and disease sequelae such as delirium. Delirium, an acute mental status change often associated with acute medical events, affects 1 in every 5 NH residents who experience an acute illness and is associated with cognitive decline immediately following the episode [24]. Delirium also affects as many as 70% of NH residents with CI/dementia [25] and is associated with a fourfold increased risk of death [26]. Delirium is also difficult to detect as older adults with dementia frequently present with nonspecific symptoms such as inattention, disorganized thinking and an altered level of consciousness [27]. An estimated 32% of delirium cases may go unrecognized by physicians, attributed instead to depression, psychosis or a worsening of dementia symptoms [27].

Because the presentation of these destabilizing events are different and subtle (agitation, anxiety, restlessness and changes in ambulation) [28] novel methods are needed to continuously assess for changes [29–33]. In preliminary work, our team identified a potentially unique method of continuous, objective, automated assessment that may identify NH residents who have increased risk for falls or have an acute health event. Using a real time locating system (RLTS), the ambulatory patterns of 26 residents were followed continuously for up to 8 months [34]. We found continuous ambulation (count of uninterrupted walking for at least 60 s where walking was not separated by at least 30-s nonambulatory intervals before and after the episode) (OR = 1.02; p ≤ 0.001) is associated with a fall within the 4-week interval in which the change was noted. The distance ambulated measure had fair sensitivity (0.74) and specificity (0.66) in predicting a fall (AUC = 0.70). Over the course of a week, fallers totalled 0.31 more miles ambulating in paths than non-fallers. Examining the cutoff that maximizes the sensitivity and specificity of the AUC measure, residents who fell had longer periods of continuous ambulation covering greater distances in each walking event. Non-fallers also had consistently sustained gait speeds and consistent time and distance travelled/week over the course of the study. This project builds on this preliminary work in the sense that continuous ambulation may predict falls; continuous ambulation may be one measure used to develop effective interventions that may reduce the number of falls in this patient population.

**Fig. 1:**
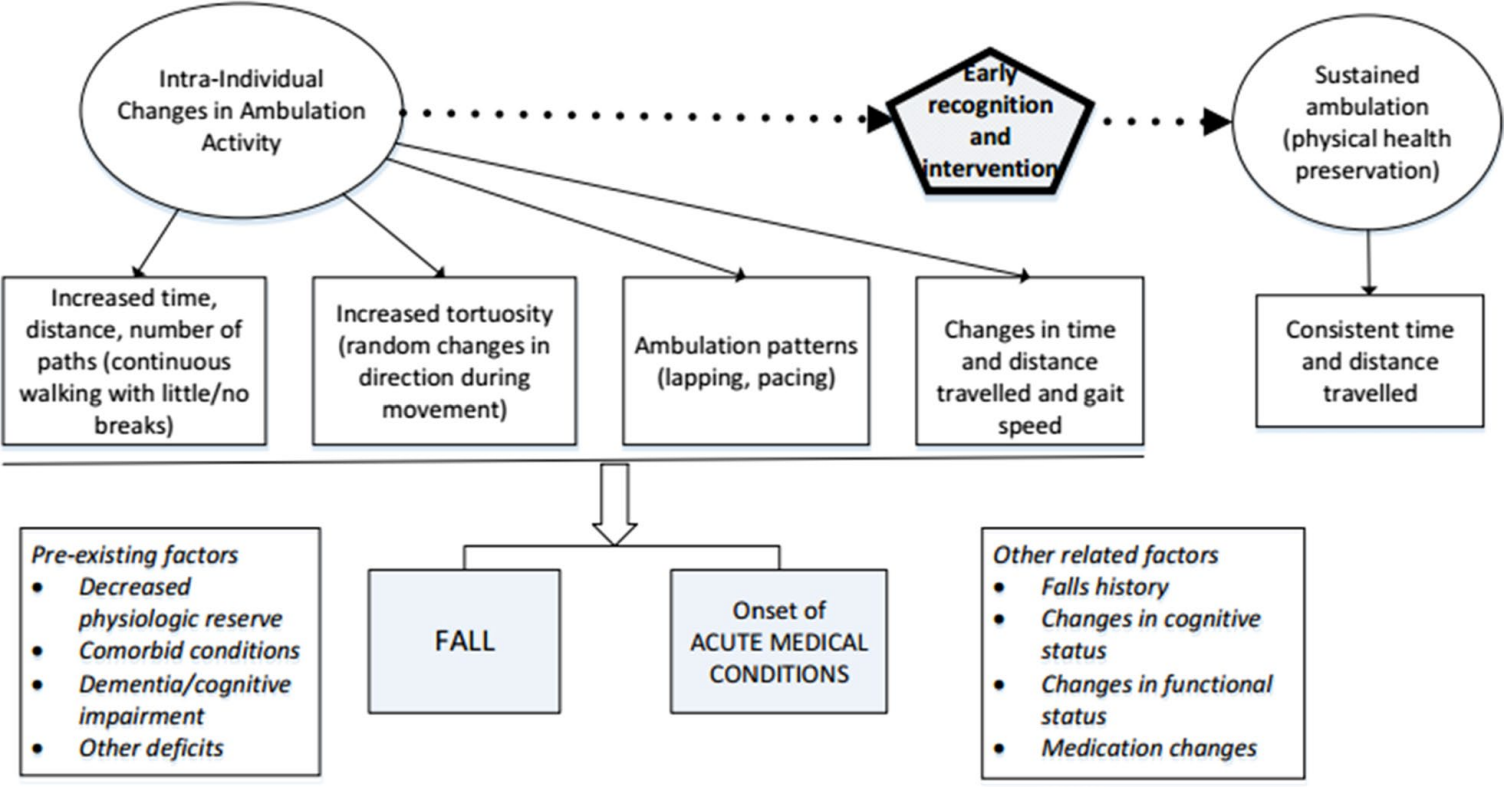
Proposed relationships between intra-individual changes in ambulation activity and acute changes in physical health

Figure 1 illustrates the types of ambulatory changes that will be examined to determine if they predict a de-stabilizing event. Based on pilot work, it is hypothesized that intra-individual changes in ambulation parameters, such as continuous ambulation, will occur as a result of the physical changes that are associated with falls and acute physical illness. The purpose of this funded study (VA RX002413-01A2) is to conduct a prospective study to test these hypotheses: H1a: Intra-individual changes in ambulation activity (e.g., path characteristics, tortuosity) will be significantly associated with a fall H1b: and the onset of acute medical conditions. The ultimate goal of this work is to determine if this type of monitoring can be used by nursing staff to quickly identify residents with CI/dementia whose risk profile has changed. This would enable a timely re-assessment of the resident by professional nursing and medical staff to provide the opportunity for treatment changes.

## Methods/design

A prospective longitudinal natural history study design will be used to determine whether changes in ambulation activity predict acute changes in physical health such as falls and acute illnesses. This study will measure but not interfere with the natural activity occurring on the units. Preliminary analyses on data collected from baseline till 1 year after the first subject is enrolled will be conducted to identify predictors of future falls and other acute medical conditions.

### Sample

Adults residing in dementia units across the three long-term care sites for whom proxies provide written consent are eligible for this study (N = 50). To be included residents must be age 55 or above, either be able to walk (with or without assistive devices) or propel with their feet. Even residents with a medical condition, or paralysis or amputation can be included in the study if they move using their own physical power. Thus, residents will be excluded from the study if they are not moving using their own physical power (e.g., in an electronic wheelchair). After the initial analysis using the total sample, subgroup analyses will be conducted to determine whether different models are needed to determine increased risk for physical health changes between those who walk versus those who propel with their feet.

Medical charts will be used to identify residents of dementia units across the three NH sites who meet inclusion criteria and then to contact the resident’s legally authorized representative (LAR) or next of kin (NOK) for consenting purposes. Because many of these residents are unable to provide consent; the study and risks will be described to LARs and NOKs either through phone calls and mailed consent documents (if the LAR is in another state) or the next time when the LAR/NOK is on the unit. After the LAR/NOK are provided the opportunity to review the documents and ask questions they will be asked to sign consenting documents. The study will also be discussed with residents with dementia whose LAR/NOK agree to consent and verbal assent (if possible) will be obtained. If there is a note in the medical chart by the geriatrician that the resident is able to make informed health decisions then informed consent will be obtained from the resident.

### Measures

#### Independent variable: ambulation activity

There are few available technologies that fulfill the criteria necessary for objectively and automatically tracking institutionalized older adults to capture changes in ambulation activity over time. This would require small, wireless, wide-area tracking, with no line of sight issues and good accuracy. This project uses Ubisense RTLS ultra-wideband radio frequency identification device (UWB RFID) [35] as this technology has been applied to several health care applications including objective measures to detect falls among institutionalized older adults [36], controlling or managing patient infection risks [29], and tracking institutionalized patients with dementia with a high degree of accuracy [37]. Most importantly for this project, the Ubisense RTLS system has been used to examine changes in the quantity of ambulation activity [38]. Other studies have similarly used this RTLS system to show a relationship between the quality of ambulation activity—or tortuosity (random changes in direction during movement) and fall risk, correlating stride time variability with random changes in direction and movement [33]. This RTLS uses compact wireless tags that can be worn by the resident to non-invasively track their movement around long-term care facilities 24/7 in real-time. These tags emit a UWB radio signal that is triangulated by multiple sensors mounted on the walls throughout the long-term care facility, based on x and y coordinates, recording resident location and time of day and storing this information for later analyses [36, 39]. UWB RFID is similar to passive RFID in most aspects, however the UWB tag itself is powered giving it a wider capture range making the spatial locating resolution of the system within 6 inches of real location, versus 36 inches or more for a typical passive RFID system.

The RTLS used here requires data smoothing to improve the location’s precision [40]. With a RTLS there are two primary issues: noise and jumps. With regard to noise, even when sedentary for several hours, the active RTLS tag continues to log motion—especially if the resident continues to move their limb where the tag is located—producing continuous movement that can artificially inflate ambulation activity measures. The location of the resident will also jump—sometimes putting a path through a wall—if the tag sleeps due to a long period of inactivity and then wakes due to resident movement. Previous work further details smoothing data to improve location precision [31]. Briefly, RTLS data are smoothed using a 5-s moving average time window and then a threshold of 0.7 m of movement is applied. This creates a stable series of coordinates, resembling the observed resident ambulation. To manage the jumps in data, when computing a day’s motion, distance and time are accrued only when time between points are less than 30 s. These algorithms are custom coded using Python and PHP [31]. After smoothing RTLS data, it is reduced to hourly, daily and weekly measures of ambulation activity (e.g., taking the average for each measure over the course of 7 days).

To capture variations in motor performance and distinguish normal variations from ambulation activity that may lead to a fall or signal the onset of an acute medical condition, multiple indices associated with the quality and quantity of ambulation activity will be measured using the RTLS data (see Table 1). This project will utilize ambulation activity data collected by a wrist-worn RTLS system for up to 2 years.

**Table 1:** The independent variables of interest in this study

Variables	When measured	How measured	Reliability and validity
Ambulation activity	Continuously; 6 times a second on each resident by a RTLS technology; triangulated location and motion data from wristband worn by resident and sensors mounted throughout CLC’s	Path characteristics: 1) Time spent walking in a path (at least 60 s of uninterrupted walking separated by at least 30 s of non-ambulatory intervals before and after the path) 2) Distance covered in a path (distance, in miles, where there is at least 60 s of uninterrupted walking) 3) The number of paths in a week (count)	1) & 2) Spearman correlation with Tinetti Gait subscale (0.32–0.35) and Tinetti Balance subscale 0.37–0.40 (unpublished data) − 95% concordance in accuracy in ambulatory path, correct location, accurate time with direct observation (observational study, unpublished data)
		Tortuosity (random changes in direction during movement measured by deviation from a straight line measured from 0–1)	Spearman correlation with stride-time variability measured by a Gait–Rite mat (0.30)^2^ Spearman correlation with Mini-Mental State Exam (− 0.47)^2^
		1) Time (minutes) and 2) distance walking (miles)	Spearman correlation with Tinetti Balance subscale 1) (0.11–0.40) Tinetti Gait subscale 2) 0.35 (unpublished data)
		Gait speed	Spearman correlation with the Tinetti Performance Oriented Mobility Assessment (0.39) (unpublished data)
		Lapping and pacing patterns first identified by hand-coding of 2-D visualizations (e.g., gif files), then using Rubine classifiers to identify patterns	Inter-rater reliability for observations of these patterns in this setting and population (0.89)

#### Dependent variable: falls and other acute events

Falls and other acute events (see Table 2) will be captured by a weekly medical chart review for each resident. Any evidence of a new fall or condition will trigger a Significant Event Audit [41] where information about the event will be collected from a variety of sources including medical record data, nursing and allied health care staff, medical staff and others who may have witnessed or assessed the event. The American National Standards System of Injury [42] will be used to capture any injury from skin bruises/scrapes to fractures as well as the nature of the injury (what was injured); source of the injury (what caused the injury); the accident type (what caused the event); the event(s) surrounding the injury; time and place of injury occurrence; and disposition (e.g., temporary placement in hospital or rehabilitation). Acute medical conditions will be captured using a similar process. Because delirium is often missed as an acute medical condition, the Short Confusion Assessment Method (CAM) [27] will be administered by research staff bi-weekly with a functional status (FS) assessment. If positive, the Richmond Agitation and Sedation Scale (RASS), which provides a measure of level of consciousness, will be administered [43].

**Table 2:** The dependent variables of interest in this study

Variables	When measured	How measured	Reliability and validity
Fall	Weekly	Medical chart review and a significant event audit [41]	Widely used formal analysis of events that affect patient care [41]
		The American National Standards System [42]—completed with Staff Interview	Widely used industry standard to monitor injuries and illnesses in the workplace; [58, 59] also used by researchers to code fall-related injuries and fall context among older adults with dementia [60]
Acute Medical Conditions (UTI’s, delirium, pneumonia, influenza, other acute illnesses and infections)	Weekly except for delirium which will be assessed twice a month	Medical chart review	–
		Delirium will be measured by: the short confusion assessment method [27]	Inter-rater reliability (0.70–1.00) [61] kappa = 0.70 [62]
		Richmond Agitation and Sedation Scale (RASS) [43] if the short CAM is positive	Inter-rater reliability (0.92–0.98) kappa (0.64–0.82) [63] Spearman correlation with other sedation scales (0.78) [63]

#### Covariates, clinical variables

Clinical variables (see Table 3) including a falls history, dementia diagnoses (and subtype, where available) and comorbid conditions, will be collected through medical chart review at baseline and every 6 months. We will also assess new medical diagnoses and medications each week by medical chart review to capture changes. FS will be measured by the Physical and Cognitive Performance Test for Assisted Living Facilities (PCPT ALF) [44] and the Barthel Index [45]. FS instruments will be administered at a consistent clock hour and day for each resident to reduce variability that occurs across the daytime period among residents with CI/dementia. FS assessments will be performed according to the standard practices of the instrument and take about 15 min each to complete. The PCPT ALF data will be used to assess FS changes occurring in conjunction with a fall or acute medical condition in an independent model as well as a combined model with RTLS data. Changes in cognitive status and gait and balance will be measured by the Montreal Cognitive Assessment (MoCA) [46] (administered at baseline and every 6 months) and the Tinetti Performance Oriented Mobility Assessment (administered bi-weekly) [47]. Medical chart reviews will be conducted to collect age (years), gender, and education (years).

**Table 3:** Clinical variables to be collected in this study

Variables	When measured	How measured	Reliability and validity
Falls history, comorbid conditions, medications	Baseline for all but new medical diagnoses and medications which are assessed weekly	Medical chart review	–
Functional status (FS)	Bi-weekly	FS measured by the PCPT ALF and the Barthel Index (the latter for reliability and validity purposes) [45]	PCPT ALF = test–retest reliability (≥ 0.60) [44] Spearman correlation with the Barthel, the Functional Independence Measure and the MDS Resident Assessment Instrument (≥ 0.70; unpublished data) Barthel = Inter-rater reliability 0.89; [64] Pearson (r) 0.50; Alpha coefficients 0.62–0.80
Cognitive status	Baseline and every 6 months	Changes in cognitive status will be measured by the Montreal Cognitive Assessment (MoCA) [46] Dementia diagnosis and subtype	Test–retest reliability = 0.92 [46] Inter-rater reliability = 0.81 Internal consistency = 0.83
Gait and balance	Bi-weekly	The Tinetti Performance Oriented Mobility Assessment [47]	Test–retest reliability = 0.72–0.86 [65] Inter-rater reliability = 0.84 [66]

#### Data collection protocol

Previous work details the protocol for the RTLS sensor set up in a long-term care facility [31]. Briefly, this requires mounting sensors in the corners of the unit where tracking is desired and calibrating RTLS tags (embedded in wristbands), and connecting (wirelessly) to a server. Each resident has their own unique identification number. The resident’s location is determined by x and y coordinates which are compared to a known sensor location, and transmitted to the server on the unit [48]. SmartFactory Ubisense software [31] writes x, y coordinates to a SQLite database [49] which is exported to IBM SPSS.

#### Data management

To merge fall, acute medical condition, RTLS, clinical data and demographic characteristics accurately for each resident, each form of record in IBM SPSS will include (1) variables to uniquely identify the resident, (2) the source of the data, (3) site, (4) date, and (5) data collector. Bi-weekly data will be entered into a data management program (IBM SPSS) on an encrypted password-protected desktop computer located in a locked office. Ambulation, fall and acute medical condition data will be transferred bi-weekly from the server. RTLS data will be reviewed bi-weekly to minimize missing data. If the resident has a fall, acute medical condition or other event during the initial 3-months enrolled in the study, a new 3-month period will begin until the resident can establish an ambulation activity baseline that is event-free across the 3-month period. To re-enter the study after any event the resident must be relocated back to the dementia unit, be a full-time resident (not in a rehabilitation bed) and be able to move using their own physical power. After baseline is established, residents who experience a fall or acute medical condition or other event will be retained in the study. Data that do not meet these criteria will be removed from the analytic dataset.

### Statistical analysis

Data management and descriptive statistics will be performed using the IBM SPSS Statistics 25 (Chicago, IL) statistical package. Hierarchical Linear Modeling (HLM; Scientific Software International, Lincolnwood, IL) statistical techniques will be used to examine relationships between ambulatory activity and acute changes in physical health. To examine intra-individual changes in ambulation activity over time a baseline will be established by examining each resident’s weekly mean, median and standard deviation on each of the ambulation activity measures for each resident during their first 3 months of study enrollment. The average of these descriptive statistics will be their baseline value. If the resident has an event within this 3 months period they will be re-entered into the study until a baseline can be established.

To examine how intra-individual changes in behavior associated with ambulation activity are associated with a fall and may indicate the onset of acute medical conditions, ambulation activity indices will be examined in two HLM models to examine individual and aggregate levels of data over time [50]. This multilevel modeling technique conceives of each resident as having their own regression equation but incorporates each week’s measure of ambulation activity simultaneously in the same model. The models are a series of nested models, one for each level of the hierarchy. At the first level, each resident’s trajectory of change in fall/acute medical condition risk will be represented as a function of person-time-specific parameters (e.g., ambulation activity indices) plus random error. The second level statistically models individual variations in growth parameters (e.g., demographic characteristics) across a population of persons. Multilevel models account for between-subject heterogeneity and within-individual correlations and model cluster-induced errors in the intercepts and coefficients to increase the efficiency of the estimates. These are good tools for analyzing repeated measures data from single subject studies [51]. The software HLM can fit multilevel models for both continuous outcomes such as the FS score and nominal outcomes such as the indicator for falls. In addition to HLM, we will apply the PROC MIXED, PROC NLMIXED and PROC GLIMMIX in SAS for the same multilevel modeling to (1) validate results from HLM and (2) use additional features of multilevel modeling implemented in SAS such as the built-in correlation structures and model selection criteria.

Subsequently, we will run these models using data collected from paper and pencil gait and balance tools to determine whether the new method of continuously monitored ambulation is superior in predicting a fall and/or acute medical conditions. If the new model is superior, gait and balance variables will be used to determine whether they strengthen predictions when used as moderator variables. Physiological fatigue may also affect the relationship between ambulation activity and falls [38]. While not a focus of this study, we will explore this relationship by conducting individual analyses focused on resident path distance 1–2 days immediately prior to the fall. If physiological fatigue is associated with falls, path distance would increase until a decrease occurs 1–2 days prior to the fall, indicating fatigue, and increased fall vulnerability.

Finally, we will utilize machine learning methods such as random forests (RF) to determine the most robust predictors of acute events from the ambulation data. RF is a class of statistical learning models originated from classification trees. RF models are data-driven and robust. RF models generate the relative importance measures of all the predictors which allow users to select the most influential predictors. Partial least squares (PLS) models are powerful predictive models for high dimensional correlated dependent variables regressed on high dimensional independent variables. PLS also permits the use of both manifest and latent variables with complex pathways. PLS also generates variance-in-projection measures which are measures of relative importance of predictors. RF is implemented in the R package *randomForest* and PLS is implemented in SAS PROC PLS. Machine learning approaches will be applied to learn from the high-dimensional raw data from ambulation activity, patterns, clinical variables, and demographic characteristics to identify the most influential predictors and combinations of predictors and to build accurate predictive models. We can add clinical data as additional features to our ambulation pattern classifier to determine if it performs with higher accuracy than with x, y position data alone. We will use the same general design strategy as we extract more features from the additional measurements.

#### Sample size and power calculation

This study has the sample size required to detect the smallest meaningful difference in standard deviation units (Δ): [52]. The sample size formula is

$$m = \frac{{2(Z_{\alpha } + Z_{Q} )^{2} \left\{ {1 + (n - 1)\rho } \right\}}}{{n\Delta^{2} }}$$

where *m* is the number of subjects per group, $\alpha$ = 0.05, Q = 1 − P, P is the power of the test and is set to 0.8 and $n$ is the number of repeated measures and $n$ = 2 in our planned study, ρ is the correlation between the repeated measures. From previous work [38], the effect sizes for time spent walking, walking distance and gait speed ranged from 0.06 to 0.57; the effect sizes for path patterns ranged 0.31–0.64; and the effect sizes for the Barthel Index instrument ranged 1.0–1.2. We simulated the autocorrelation ρ to be small (0.05), moderate (0.25) and large (0.8). The required sample sizes for different combinations of operating parameters are shown in Table 4. Results from this simulation show that with the current repeated measures design and a sample size of N = 50, we can detect a small effect size around 0.15 even with high autocorrelation for the repeated measures.

**Table 4:** Required sample sizes for proposed study

ρ	∆		
	0.05	0.15	0.20
0.05	66	7	4
0.25	161	17	10
0.80	421	46	26

## Discussion

Falls and acute health changes such as pneumonia, UTIs and upper respiratory infections are associated with declines in physical function, hospitalization, and death among NH residents. Persons with dementia have atypical presentations of acute health changes often presenting with delirium superimposed on dementia, functional decline, or falls [53]. The findings from this study will be used to understand ambulation-related behavioral changes and how these can identify, with high sensitivity and specificity, older adults at an increased risk for falls and the onset of acute medical conditions—to ultimately develop mechanisms that trigger further assessment and modifications to individual plans of care. To this end, we will use a combination of data acquisition and analysis to understand how to use real-time data from the RTLS system to detect worrisome ambulatory changes and learn how to best communicate this information to nursing staff to initiate expert assessments and evidence-based individualized interventions.

First, we will work with biomechanical/fall, statisticians and clinical experts to identify the best ambulatory patterns to predict destabilizing events. Then we will ‘optimize’ our conceptual model, adding underlying physiological mechanisms that explain the ambulatory changes [54]. A qualitative study will be conducted over the course of 2 days, six focus groups will be conducted (two at each site), lasting approximately 1 h each. At each site we will schedule one session in the morning and one session in the evening so nursing staff from various shifts can attend. These clinical staff focus groups will explore and identify processes to effectively alert nursing staff to the detected changes in a resident. These qualitative groups will also be used to develop and integrate evidence-based clinical decision and treatment trees to provide highly individualized care to an at-risk resident. In addition, by including the nursing staff in the development of the notification processes as well as the development of a decision-tree for individualized interventions the nursing gain a sense of commitment to the process.

The Department of Veterans Affairs (VA) is primed to conduct this research for several reasons. First, the VA has knowledgeable RTLS researchers focusing on multiple geriatric patient populations across several outcome areas [30, 31, 33, 39]. For example, a RTLS has been used to detect a fall in real-time [36] and track the potential spread of multi-drug resistant organisms [29]. Thus, there is a community of VA researchers who will be able to utilize and distribute findings from this study. In addition, Patient Aligned Care Teams (PACTs) which are based on the patient-centered medical home model [55], focus on care coordination, health care access, providing comprehensive care, and integrating psychosocial and environmental determinants of health [56]. These PACTs enable earlier adoption of study findings—namely how behavioral information may be used to preserve the functional status of NH residents. In addition, the VA has expanded PACT to reach more specific populations; for example, the Geriatric Patient Aligned Care Teams, which provide health care for a subset of older Veterans with chronic disease, functional dependency, cognitive decline, and psychosocial challenges [57]. Finally, there are about 133 VA NH’s in the United States that utilize this team and patient-centered approach to care and 39 of these facilities have some form of a RTLS (3 sites have the full RTLS implementation described here). While clinicians have not yet incorporated the use of a RTLS in patient care there is the potential for this study findings to be disseminated at additional test sites and translated into practice for clinicians to use this technology in order to tailor care plans for residents at most risk for falls and the early detection of acute events.

## Data Availability

Not applicable. This manuscript currently does not contain any data.

## Abbreviations

RTLS: real-time locating system
UTI: urinary tract infection
NH: nursing home
CI: cognitive impairment
CNA: certified nursing assistant
LAR: legally authorized representative
NOK: next of kin
UWB RFID: ultra-wideband radio frequency identification device
CAM: Short Confusion Assessment Method
FS: functional status
RASS: Richmond Agitation and Sedation Scale
PCPT ALF: Physical and Cognitive Performance Test for Assisted Living Facilities
MoCA: Montreal Cognitive Assessment
HLM: Hierarchical Linear Modeling
RF: random forests
PLS: partial least squares
VA: Department of Veterans Affairs
PACTs: patient aligned care teams
